# Biosynthesis of Nanomaterials by *Shewanella* Species for Application in Lithium Ion Batteries

**DOI:** 10.3389/fmicb.2018.02817

**Published:** 2018-11-21

**Authors:** Tae-Yang Kim, Min Gyu Kim, Ji-Hoon Lee, Hor-Gil Hur

**Affiliations:** ^1^School of Earth Sciences and Environmental Engineering, Gwangju Institute of Science and Technology, Gwangju, South Korea; ^2^Pohang Accelerator Laboratory, Pohang University of Science and Technology, Pohang, South Korea; ^3^Department of Bioenvironmental Chemistry, Chonbuk National University, Jeonju, South Korea

**Keywords:** *Shewanella* species, metal-reducing bacteria, biogenic nanomaterials, chalcogenides, lithium ion batteries, *in situ* XAFS analysis

## Abstract

Nanomaterials exhibit extraordinary properties based on their size, shape, chemical composition, and crystal structure. Owing to their unique properties nanomaterials are preferred over their bulk counterparts for a number of applications. Although conventional physical and chemical routes were established for the massive production of nanomaterials, there are some drawbacks such as environmental burden and high cost that cannot be disregarded. Recently, there has been great interest toward the green synthesis of inorganic nanomaterials. It has been reported that dissimilatory metal reduction by microorganisms is a cost-effective process to remediate toxic organic and inorganic compounds under anaerobic conditions. Particularly, members of the *Shewanella* genus have been utilized to produce various biogenic nanomaterials with unique micro/nanostructured morphologies through redox transformations as well as to remove harmful metals and metalloids in eco-efficient and environment-friendly methods under ambient conditions. In the present mini-review, we specifically address the active utilization of microbial respiration processes for the synthesis of novel functional biogenic nanomaterials by the members of the *Shewanella* genus. This biosynthetic method may provide alternative approaches to produce electrode materials for sustainable energy storage applications.

## Introduction

Nanostructured materials are indispensable to modern life, and they are critical components of multiple industrial and consumer products, including daily necessities. For example, metallic nanoparticles (NPs) with tunable size, shape, and crystal structure have been applied in optical, electrical, and medical instruments. Over the years, researchers in academia as well as industries have investigated several methods for the synthesis of NPs, and various conventional physical and chemical methods have been established. However, most methods are not sustainable due to requirement of harmful or expensive chemicals, extreme temperatures and pressures during manufacturing processes, increased risk to environment and public health, and high energy consumption and waste generation (Cueva and Horsfall, 2017). Biotechnological routes such as the use of bacteria, fungi, and plant-extracts for synthesizing NPs are attractive alternative green approaches that utilize environment-friendly precursors and processes under mild conditions (Li et al., 2011; Pantidos and Horsfall, 2014; Prasad et al., 2018). In general, interdisciplinary studies between bionanotechnology and materials science are expected to accomplish major breakthroughs to resolve urgent environmental challenges such as industrial pollution and depletion of resources and achieve sustainable development goals.

Metal-reducing bacteria are considered eco-friendly catalysts for both bioremediation and materials synthesis. In particular, the members of the *Shewanella* genus can assist in the formation of diverse metal oxides and chalcogenides (materials containing S, Se, or Te elements) through microbial respiration processes. Notably, biogenic nanomaterials with unique morphologies and crystal structures produced by the members of the *Shewanella* genus can be used as Li-ion active electrode materials for lithium ion batteries (LIBs). The search for new chemistry or materials has received considerable attention for the production of high-performance LIBs, which are essential components in electric cars, portable gadgets, and several home appliances.

To the best of our knowledge, the biosynthesis of nanomaterials through the respiration process of metal-reducing bacteria and the application of these nanomaterials in LIBs has scarcely been reported. The current review provides a comprehensive overview on evidence from the two areas of research, namely the eco-efficient production of biogenic nanomaterials by the members of the *Shewanella* genus and their possible applications as Li-ion active electrodes for use as sustainable energy storage systems.

## Dissimilatory Metal Reduction Process to Materials Synthesis

### Microbial Respiration Process

Microbial dissimilatory anaerobic respiration can transfer electrons from reduced organic to oxidized inorganic compounds, thereby facilitating the cycling of carbon and metals as well as bioremediation processes. It is well-known that the members of the *Shewanella* genus are capable of coupling the oxidation of organic acids, as electron donors, to the reduction of inorganic metals, metalloids, and radionuclides, as electron acceptors, thereby changing the valence and oxidation state of the molecules through anaerobic respiration (Heidelberg et al., 2002; Tiedje, 2002; Harris et al., 2018). Bioreduction by the members of the *Shewanella* genus was discovered through the function of extracellularly excreted flavins and bacterial nanowires (Marsili et al., 2008; El-Naggar et al., 2010; Beblawy et al., 2018). This research, initiated for the immobilization of toxic metals and metalloids in aqueous environments, has since been utilized for the production of nano-sized inorganic compounds through microbial metal reduction processes.

### Synthesis of Biogenic Nanomaterials by *Shewanella* Species

Anaerobic respiration processes provide facile synthetic strategies for the formation of unique minerals and nanomaterials under ambient conditions (Gadd, 2010). Recently, it was reported that the members of the *Shewanella* genus could produce various metal NPs such as silver (Ag), copper (Cu), palladium (Pd), iron (Fe), or manganese (Mn) oxides. *Shewanella oneidensis* MR-1 produced biogenic Ag-NPs that exhibited higher bactericidal activity compared with abiotic counterparts (Suresh et al., 2010). Kimber et al. (2018) showed that *S. oneidensis* MR-1 can synthesize Cu-NPs through bioreduction of Cu(II). The Pd-NPs produced by *S. oneidensis* MR-1 and *S. loihica* PV-4 showed significantly higher catalytic activities than the chemically produced equivalents (Wang et al., 2018; Xiong et al., 2018). In addition, the members of the *Shewanella* genus can produce iron or manganese oxides (Jiang et al., 2013; Wright et al., 2016).

**FIGURE 1:**
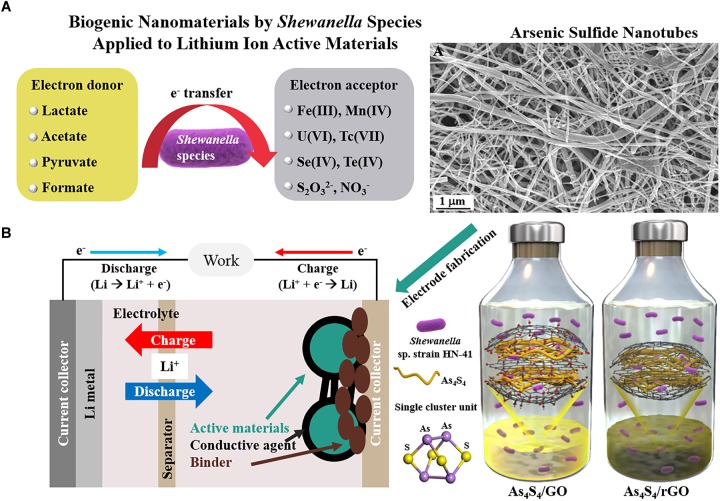
Application of biogenic nanomaterials produced by members of the *Shewanella* genus. **(A)** Microbiological respiration process and biogenic arsenic sulfide nanotubes (Reproduced with permission from *PNAS* 104 (51), 20410-20415. Copyright 2007 National Academy of Sciences, United States) and **(B)** biogenic As_4_S_4_/reduced graphene oxide applied as Li-ion active materials (Copyright 2017 Wiley. Used with permission from *Adv. Sustain. Syst.* 1(7), 1700056).

The elements sulfur (S), selenium (Se), and tellurium (Te) belong to the oxygen family, also called chalcogens (meaning “copper producing”). Chalcogenide materials have been used widely in optical devices, phase change materials, and photovoltaic applications (Kovalenko et al., 2009; Sousa, 2011; Zhou et al., 2018). Production of diverse metal chalcogenides by a number of bacteria has been reported (Jacob et al., 2016; Vena et al., 2016; Boedicker et al., 2018). The chalcogens have a high affinity toward metals, making them powerful adsorbents for bioremediation process (Ralston, 2008). For example, toxic arsenate (As^5+^) can be reduced to arsenite (As^3+^) via microbial reduction process and precipitated into arsenic sulfide nanomaterials in the presence of thiosulfate (S_2_O_3_^2−^) by using *Shewanella* sp. strain HN-41 (Figure 1A; Lee et al., 2007). The precipitation of the arsenic sulfide has also been observed using other members of the *Shewanella* genus (McFarlane et al., 2015).

Another chalcogen element, Se, can be toxic in high doses and occurs in the form of oxyanions Se(IV) and Se(VI), which can be reduced by diverse Se-respiring bacteria to insoluble elemental Se (Pearce et al., 2011; Fernández-Llamosas et al., 2017; Shoeibi et al., 2017). It was reported that biogenic Se nanospheres could be applied to capture toxic mercury to remove both heavy metals without leading secondary pollutions (Jiang et al., 2012). Interestingly, biogenic amorphous Se nanospheres can be transformed into crystalline nanowires in dimethyl sulfoxide solvent at ambient conditions (Ho et al., 2010), suggesting that biological synthesis of nanomaterials could be more tunable and controllable with a combination of materials such as organic solvents and substrates.

The other chalcogen element, Te, has been exploited for numerous applications, leading to environmental issues due to high-toxicity of its oxyanions Te(IV) and Te(VI) (Ba et al., 2010). *S. oneidensis* MR-1 can utilize toxic soluble tellurite as electron acceptor, resulting in the formation of Te nanorods. It has been reported that needle-shaped crystalline Te nanorods were intracellularly or extracellularly formed depending on the reaction conditions (Kim et al., 2012, 2013). In short, the chalcogenide nanomaterials formed as the by-products from their bioremediation processes, with controllable morphologies, can be a valuable precursor for multiple applications.

## Application of Biogenic Nanomaterials by *Shewanella* Species for Lithium Ion Batteries

LIBs are widely used as energy storage devices for portable electronics such as smartphones, home electronics, medical devices, and defense systems, mainly due to their light weight, high energy density, low memory effect, and low self-discharge rate. Interdisciplinary research can help with the current challenges of making sustainable LIBs with more eco-efficient processes (Larcher and Tarascon, 2015). Nanomaterials are expected to enhance the battery performance owing to short diffusion length for Li-ion traveling and high surface area (Aricò et al., 2005). Various nanostructured metal oxides and sulfides have gained attention as potential Li-ion active electrode material for LIBs (Wu et al., 2012; Xu et al., 2014). Furthermore, the use of nanomaterials synthesized by various nanobiotechnological routes as electrode materials in LIBs has been reviewed (Zhang et al., 2015). For example, the surfaces of modified viruses or bacteria have been utilized as biotemplates for LIB electrode materials. However, the low yield and complicated synthetic processes have hindered the industrial application of viral templates. In contrast, biomineralized iron oxides, produced by metal-oxidizing bacteria, have been used extensively as precursors for Li-ion active electrode materials (Miot et al., 2014). The LIB cell is composed of an anode, cathode, electrolyte, and a separator (Figure 1B). In a commercial cell, the cathode part is a lithium cobalt oxide and the anode is usually graphite. To test Li-ion storage capability of biogenic nanomaterials, they can be reacted with lithium metal. For the preparation of the LIB cell, biogenic nanomaterials as Li-ion active materials have to be physically mixed with conductive agent and binder to increase conductivity and mechanical stability between active materials, conductive agent, and current collector (Korthauer, 2018). Thus, nanostructured iron oxide and metal chalcogenide NPs produced by the members of the *Shewanella* genus have gained considerable interest as new materials or chemistry for LIBs because S, Se, or Te have already been extensively investigated as Li-ion storage materials (Bruce et al., 2012; Seo et al., 2015; Eftekhari, 2017).

**FIGURE 2:**
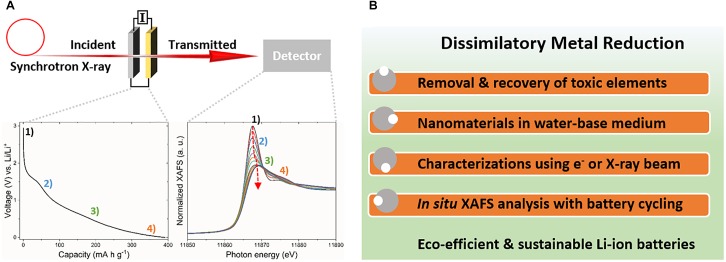
Methods for research on biogenic nanomaterials. **(A)** *In situ* X-ray absorption spectroscopy during charge-discharge processes and **(B)** collaborative research activities for microbiological synthesis and application of biogenic nanomaterials.

Biogenic arsenic sulfide (As_4_S_4_) nanomaterials have been applied as photoconductive devices (Lee et al., 2007) or field-effect transistors (McFarlane et al., 2015). Moreover, the unique cage-like molecular structure of the biogenic As_4_S_4_ formed by *Shewanella* sp. strain HN-41 was adopted for Li-ion active electrode materials (Kim et al., 2017). Intrinsic low conductivity of the biogenic As_4_S_4_ was improved by *in situ* microbial reduction of graphene oxide (Salas et al., 2010; Wang et al., 2011), a usual tactic to increase electrical conductivity (Raccichini et al., 2014). As a second example, biogenic Te nanorods formed by *S. oneidensis* MR-1 have an integrated network of closely packed atomic wires. The empty volume space between the atomic wires of the Te nanorods was anticipated as a unique Li-ion storage material (Kim et al., 2015). Thermal carbonization of intracellular or extracellular biogenic Te nanorods with bacterial cells provided encapsulation of the Te nanorods in the carbon matrix, which increased electrical conductivity and enhanced battery performance. Interestingly, the intracellular Te nanorods exhibited different phase transition during the carbonization process compared with the extracellular Te material, revealed as distinct voltage profile characteristics. Distinct Li-ion storage mechanism of biogenic nanomaterials can be investigated during battery charge-discharge cycles via various *in situ* methods such as X-ray diffraction or X-ray absorption techniques (Figure 2A; Hapuarachchi et al., 2018). The structural changes of the biogenic As_4_S_4_ or Te nanomaterials during charge-discharge processes suggest the possible application of the biogenic nanomaterials for high-performance Li-ion active anode systems.

Thus, research has enabled the removal of the toxic water-soluble As or Te from aqueous environments through immobilization into insoluble As_4_S_4_ or Te precipitates that have lower bioavailability. Furthermore, these precipitated As_4_S_4_ or Te nanomaterials have been successfully revalorized in field-effect transistors (McFarlane et al., 2015) and LIBs (Kim et al., 2017). However, the issue of recycling these batteries and transistors still needs to be addressed. Compared with the well-established conventional recycling of lead-acid batteries, there are currently no procedures in place for the recycling of spent LIBs (Gaines, 2014). However, bioleaching appears to be an environment-friendly way to recover valuable compounds such as Co, Ni, or Au, as well as As or Te in spent LIBs (Biswal et al., 2018). Apart from the issue of recycling, it is desirable to synthesize valuable nanomaterials using non-toxic resources. Iron oxide nanomaterials biosynthesized by metal-oxidizing bacteria have been applied as electrode materials (Miot et al., 2014). Moreover, it was reported that iron phosphate nanomaterials produced by *Shewanella* species have been successfully applied as electrode materials in LIBs (Kageyama et al., 2015).

## Future Perspectives

The synthesis of nanomaterials by dissimilatory metal-reducing bacteria and their application as Li-ion active electrode materials should involve interdisciplinary collaborative research with following aims (Figure 2B):

(1) Development of eco-efficient and environment-friendly bioremediation processes for the removal of harmful heavy metals, metalloids, and radionuclides.
(2) Green synthesis for low-cost and environment-friendly biogenic nanomaterials.
(3) Characterization of the size, morphology, and crystal structures of the prepared biogenic nanomaterials using electron and X-ray analysis.
(4) Application of biogenic nanomaterials as Li-ion active electrode materials and *in situ* synchrotron X-ray analysis during charge-discharge processes to reveal their Li-ion storage mechanism.

One of challenges for the application of biogenic nanomaterials is their inhomogeneous morphologies or chemical compositions compared with the chemically synthesized counterparts. In addition, the presence of large quantities of organic compounds such as bacterial cell debris or excreted proteins may reduce the electrical conductivity and hinder a direct application of biogenic nanomaterials. Thus, post-treatment might be a requirement to improve the applicability of biogenic nanomaterials or to enhance their physicochemical properties.

## Concluding Remarks

The exploitation of microorganisms for bioremediation has garnered considerable research interest. The anaerobic respiration process can be used to transform toxic elements into sparingly soluble forms with low bioavailability and toxicity. This microbiological transformation process can also provide an alternative green chemistry approach for the biosynthesis of nanomaterials for use in sustainable Li-ion storage systems. The investigation and application of the biogenic nanomaterials produced by members of the *Shewanella* genus, can provide valuable insights into the ecological aspects of microbe–metal interaction and yield important milestones for the development of eco-efficient and sustainable Li-ion storage technologies, while simultaneously reducing environmental burden.

## Author Contributions

T-YK wrote the draft of the manuscript as the first author. MGK and H-GH contributed to the conception and design of the work. J-HL revised the manuscript critically for important intellectual content. H-GH provided approval for the publication as the corresponding author.

## Conflict of Interest Statement

The authors declare that the research was conducted in the absence of any commercial or financial relationships that could be construed as a potential conflict of interest. The reviewer VE-B and handling Editor declared their shared affiliation.
